# A new species of *Torrestrongylus* (Trichostrongylidae, Anoplostrongylinae) from *Macrotus waterhousii* (Chiroptera: Phyllostomidae) in Central Mexico

**DOI:** 10.1051/parasite/2015029

**Published:** 2015-10-29

**Authors:** Juan Manuel Caspeta-Mandujano, Jorge Luis Peralta-Rodríguez, María Guadalupe Galindo-García, Francisco Agustín Jiménez

**Affiliations:** 1 Laboratorio de Parasitología de Animales Silvestres, Facultad de Ciencias Biológicas, Universidad Autónoma del Estado de Morelos Av. Universidad No. 1001, Col. Chamilpa C.P. 62210 Cuernavaca Morelos Mexico; 2 Centro de Investigaciones Biológicas, Universidad Autónoma del Estado de Morelos Av. Universidad No. 1001, Col. Chamilpa C.P. 62210 Cuernavaca Morelos Mexico; 3 Facultad de Ciencias Agropecuarias, Universidad Autónoma del Estado de Morelos Av. Universidad No. 1001, Col. Chamilpa C.P. 62210 Cuernavaca Morelos Mexico; 4 Department of Zoology, Southern Illinois University 62901-6501 Carbondale Illinois USA

**Keywords:** Nematode, Parasite, *Torrestrongylus tetradorsalis*, *Macrotus waterhousii*, Chiroptera, Mexico

## Abstract

A new species of nematode, *Torrestrongylus tetradorsalis* n. sp., is described herein, based on specimens recovered from the small intestine of the leaf-nosed bat, *Macrotus waterhousii*, from the Biosphere Reserve “Sierra de Huautla” in the state of Morelos, Mexico. The new species is included in *Torrestrongylus* because it features a bursa of the type 3 – 2, a divided cephalic vesicle with an anterior half in the shape of an umbrella, and a posterior widened half. The new species can be distinguished from the only other congener *T. torrei* Pérez-Vigueras, 1935 by four key features: first, by the absence of cervical alae in both males and females; second, by the relatively longer second half of the cephalic cap; third, by the configuration of the dorsal ray, that does not have a medial terminal ray, and finally, by the structure of the spicules. This is the second species in the genus, previously known from bats of the families Phyllostomidae and Molossidae in Cuba, and now in Mexico.

## Abstract (fr)

Une nouvelle espèce de nématode, *Torrestrongylus tetradorsalis* n. sp. est décrite ici, à partir de spécimens collectés dans l’intestin grêle du chiroptère Phyllostomidae *Macrotus waterhousii*, de la Réserve de la Biosphère « Sierra de Huautla » dans l’État de Morelos au Mexique. La nouvelle espèce est incluses dans *Torrestrongylus* car elle possède une bourse de type 3-2, une vésicule céphalique divisée avec une moitié antérieure en forme de parapluie et une moitié postérieure élargie. La nouvelle espèce est distinguée de la seule espèce congénère *T. torrei* Pérez-Vigueras 1935 par quatre caractéristiques clés ; premièrement l’absence d’ailes cervicales chez les mâles et les femelles ; deuxièmement, la seconde moitié de la capsule céphalique relativement plus longue ; troisièmement, la configuration du rayon dorsal, qui ne possède pas de rayon médian terminal ; finalement, par la structure des spicules. Ceci est la deuxième espèce du genre, déjà connu des chauves-souris des familles Phyllostomidae et Molossidae à Cuba, et maintenant au Mexique.

## Introduction

Species in Anoplostrongylinae Chandler, 1938 (Nematoda: Molineidae) are known to infect xenarthrans and bats [7]. To our knowledge, 28 species distributed in 15 genera are known to occur in microchiropterans. The Phyllostomidae contains 57 genera [23], from which the genus *Macrotus* includes two species, the Californian leaf-nosed bat, *M. californicus* Baird, and Waterhouse’s leaf-nosed bat, *M. waterhousii* Gray. The distribution of the latter is disjunct, including a continental and a Caribbean range. The continental range includes areas of western and central Mexico and north-central Guatemala, whereas the Caribbean range includes islands of the Bahamas, Beata, Cayman, Cuba, Hispaniola, and Jamaica [17]. The species has not been found in the Yucatan peninsula or in the coastal plains of the Gulf of Mexico. Waterhouse’s leaf-nosed bats are cave dwellers and form groups from a few to as many as 500 individuals; they forage twice during the night, 2 h after sunset, and 2 h before sunrise. As a consequence, some consider them “gleaning” insectivores that capture prey such as crickets, grasshoppers, beetles, and sphinx moths from the ground or foliage rather than in flight [1].

During an investigation of helminth parasites of bats in central Mexico, carried out by the research team of the Laboratorio de Parasitología de Animales Silvestres of the Universidad Autónoma del Estado de Morelos (UAEM), several nematodes of the genus *Torrestrongylus* were recovered from the small intestine of Waterhouse’s leaf-nosed bats in the State of Morelos, Mexico. A detailed morphological analysis of this material revealed the presence of a new species, which is described below.

## Materials and methods

From August 2012 to July 2013, 36 Waterhouse’s leaf-nosed bats were collected by placing a mist net across the opening of the abandoned “Mina América”, located in the Biosphere Reserve “Sierra de Huautla”, municipality of Tlaquiltenango, Morelos, Mexico (18°27′47ʺ N, 99°00′54ʺ W, 1063 m). Bats were handled and killed following recommendations set forth by the American Society of Mammalogists [16], and collected under Licencia de Colector Científico FAUT – 0211 (National Collecting Permit 0211) from the Secretaria del Medio Ambiente y Recursos Naturales (SEMARNAT). Parasitological examinations were performed in freshly killed bats, following the recommendations and protocols described in Gardner and Jiménez-Ruiz [8].

A thorough helminthological examination resulted in 219 nematodes of an unidentified species of *Torrestrongylus* that were recovered from the small intestine of 26 Waterhouse’s leaf-nosed bats. The worms collected were washed in saline solution, counted, and fixed in either hot 4% formaldehyde or 70% ethanol. Nematodes were cleared with glycerin and temporarily mounted for morphological examination. Two specimens were progressively dehydrated in a graded ethanol series, dried by critical point drying using CO_2_, affixed to a SEM stub, and sputter-coated with gold palladium. Specimens were imaged on a scanning electron microscope (Hitachi S2460 N, Tokyo, Japan). All measurements provided are in millimeters except where otherwise indicated. Upon identification, all specimens were washed and stored in vials in 70% ethanol and submitted to the Colección Nacional de Helmintos (CNHE) of the Universidad Nacional Autónoma de México (Mexico City, Mexico), the Colección Parasitológica de la Universidad Autónoma del Estado de Morelos (COPAUAEM, Cuernavaca, Mexico), the Harold W. Manter Laboratory of Parasitology (HWML, University of Nebraska, Lincoln, Nebraska), and the United States National Parasite Collection (USNPC, Smithsonian Institution).

Genomic DNA was extracted, isolated, and purified from vouchered nematodes following standard protocols [10, 15]. These aliquots were used as a template to amplify the mitochondrial gene cytochrome c oxidase subunit 1 (*cox1*); the primers and thermal profile used to complete the reactions, as well as the postamplification processing of these fragments, are identical to those described elsewhere [10]. Published sequences of available Anoplostrongylinae and other strongyles were downloaded from GenBank [10], and these include *Macuahuitloides inexpectans* Jiménez, Peralta-Rodríguez, Caspeta-Mandujano, and Ramírez-Díaz, 2014 GenBank KF425296, and KF425297, HWML68495 and HWML68496, and the species described herein with the accession numbers GenBank KF425294, HWML68493 and GenBank KF425295, HWML68494. The resulting amplicons were aligned with ClustalW (http://www.genome.jp/tools/clustalw/). The model of evolution HKY + G was selected using the corrected Akaike Information Criterion as implemented in jModelTest [12]. Parsimony and maximum likelihood were used as optimality criteria to reconstruct the phylogeny [18], and a Bayesian inference to estimate the posterior probabilities of the nodes [13]. In PAUP*, the phylogeny was reconstructed by means of a heuristic search with tree bisection reconnection (TBR) branch swapping, 100 random additions of sequences, and 10 trees held at each replicate. One thousand bootstrap replicates were performed using a heuristic search in PAUP*. For the Bayesian inference, five chains were set to run for 10 million generations with resampling every 1000 iterations and a burn-in of 25% of the resulting trees. The remaining trees were used to reconstruct the consensus.

## *Torrestrongylus tetradorsalis* n. sp.

urn:lsid:zoobank.org:act:E25AC858-E5A6-441C-A5CA-4B398D656914

Type host: Waterhouse’s leaf-nosed bat, *Macrotus waterhousii* Gray, 1843.

Type locality: Mexico: Morelos State: municipality of Tlaquiltenango: Mina América, Biosphere Reserve “Sierra de Huautla” (18°27′47ʺ N, 99°00′54ʺ W).

Site of infection: Small intestine.

Prevalence and range of intensity: 72% (26 of 36 bats examined); 1–28 nematodes.

Date of collection: From August 2012 to July 2013. Holotype, allotype, and paratypes were collected in April 2013.

Etymology: The species name, *tetradorsalis*, refers to the four terminal papillae present in the dorsal ray.

Deposition of specimens: Holotype, allotype, and paratypes in the Instituto de Biología, UNAM, Mexico City (CNHE9899 – 9901); USNPC106976 – 79; COPAUAEM N-500; HWML68495 – 98.

### Description (Figs. 1–26)

**Figures 1–12.**
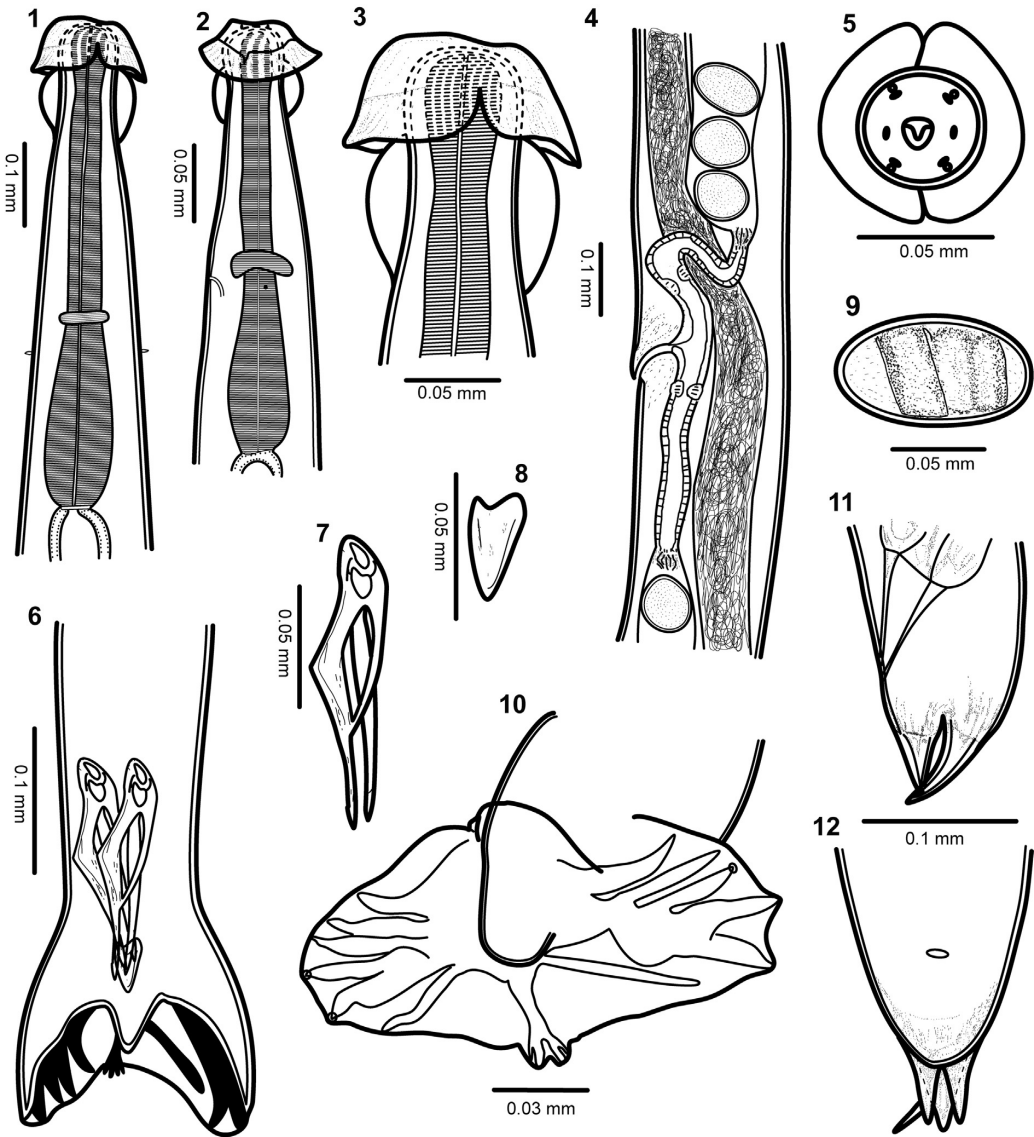
*Torrestrongylus tetradorsalis* n. sp. 1: Anterior end of male, ventral view, showing the esophagus and the relative positions of the nerve ring and deirids; note separation of cuticular projection in anterior vesicle. 2: Anterior end of female, lateral view showing the relative position of the nerve ring, deirid, and excretory pore; note continuous cuticular expansion in anterior vesicle. 3: Detail of the cephalic vesicle showing the anterior half with the lateral projections forming an umbrella and the posterior half with “handles of a pitcher” appearance. 4: Ovejector of female showing flap in anterior lip of vulva, vagina vera, infundibulum, sphincters, and uterine branches containing mature eggs. 5: Face view of male, showing arrangement of papillae and cuticular expansions of the anterior cephalic vesicle. 6: Posterior end of male, showing the relative position of spicules and gubernaculum. 7: Spicule. 8: Gubernaculum. 9: Egg. 10: Caudal bursa showing bursal arrangement and bifurcation of dorsal ray. 11: Lateral view of tail of female. 12: Ventral view of tail of female.

**Figures 13–20.**
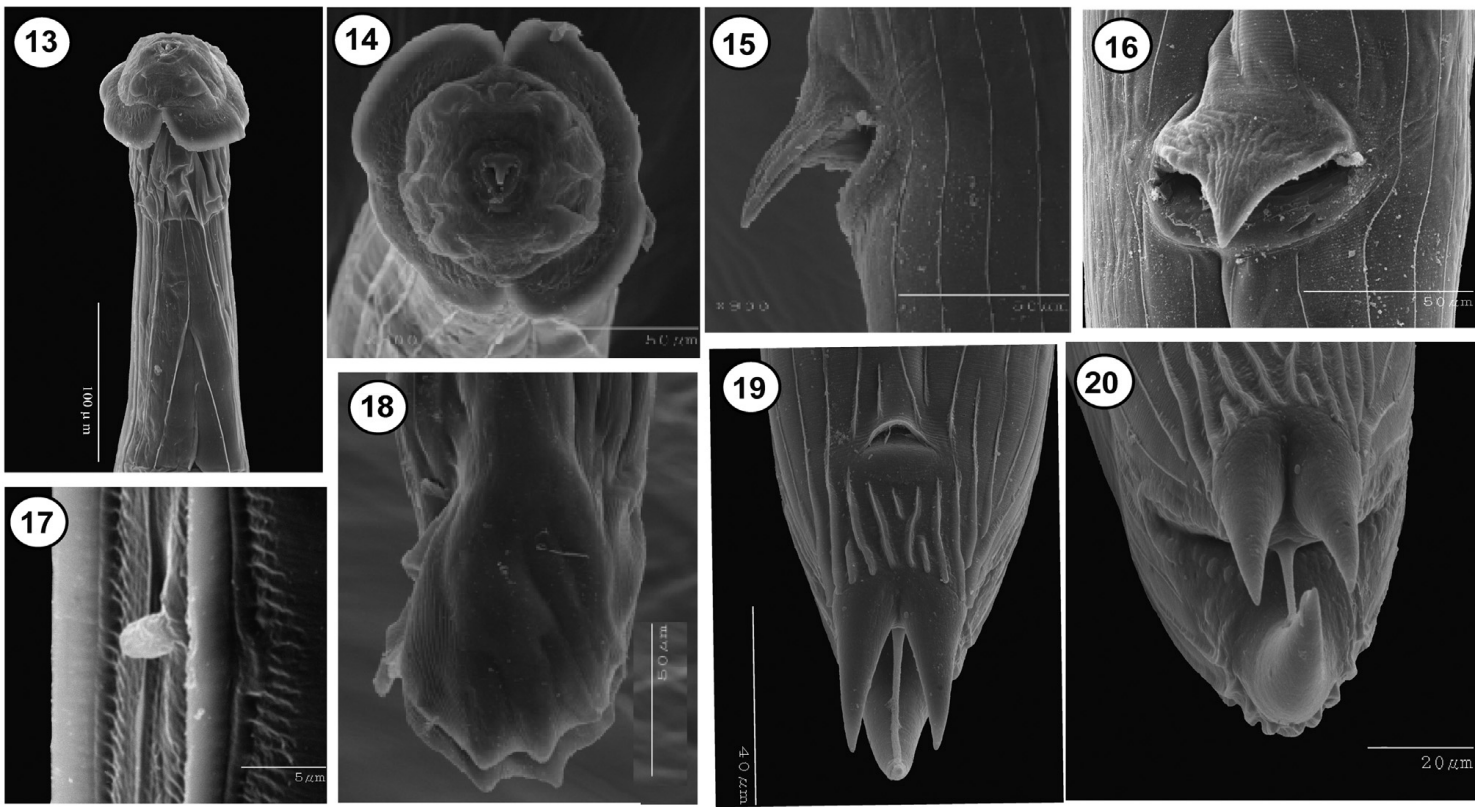
Scanning electron micrographs of *Torrestrongylus tetradorsalis* n. sp. 13: Subventral view of the anterior end, showing lateral projections of cephalic vesicle; posterior part of cephalic vesicle appears “deflated”. 14: Face view showing stoma, papillae, and amphids. 15: Detail of the vulva featuring conspicuous flap, lateral view. 16: Ventral view of vulva showing the basis of the vulvar flap. 17: Deirid. 18: Lateral view of caudal bursa, showing projections supported by bursal rays. 19: Ventral view of tail of female, featuring anus and terminal spines. 20: Subterminal view of tail of female, showing all three cuticular projections and terminal filament.

**General**: Small nematodes with synlophe armed with 32–33 ridges at midbody, ridges continuous, of equal size, supported by strut, barely emerging from cuticle (Figs. 14–16). Cephalic vesicle divided into two, anterior half in the form of an umbrella with two lateral cuticular folds, followed by a posterior half, regularly expanded without striations (Figs. 1–3, 5, 13, 14). Stoma small, triangular, with esophagorhabdion forming an inward projection or dorsal tooth (Figs. 5 and 14). Stoma flanked by amphids and endowed with four pairs of cephalic papillae; (Figs. 5, 14); small deirids simple and flattened (Fig. 17), located at midlength of esophagus (Fig. 1). Tail of male ending in subsymmetrical bursa (Figs. 6, 10, 18) and that of female conical, with three conspicuous terminal cuticular processes or tubercles, and a fine terminal thread (Figs. 11, 12, 19, 20).

**Figures 21–26.**
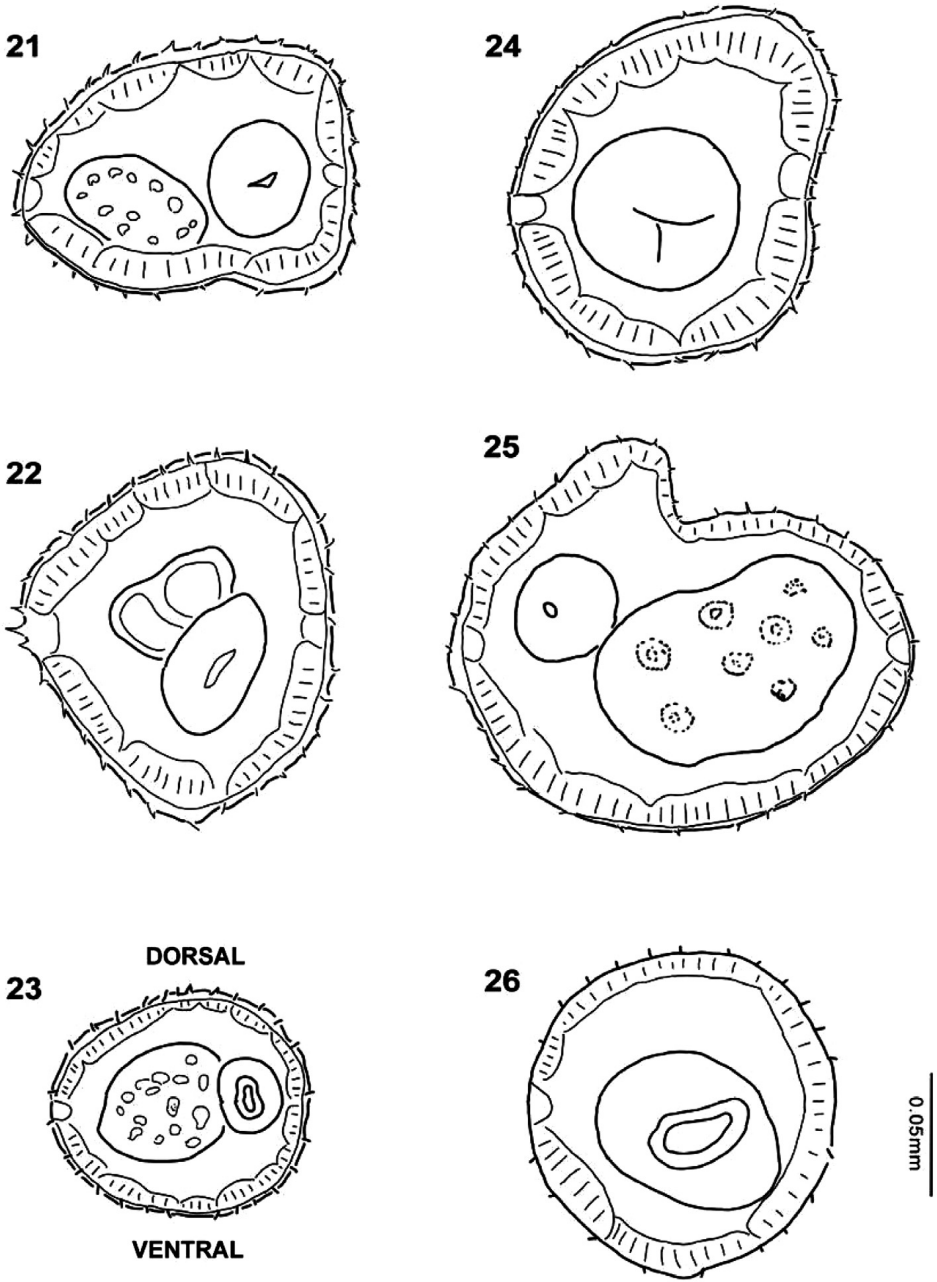
Cross section of *Torrestrongylus tetradorsalis* n. sp. Male at level of 21, nerve ring, 22 midbody and 23, posterior end of intestine. Female at level of 24, nerve ring, 25 midbody and 26, posterior end of intestine.

**Male** (based on 10 specimens; ranges, see Table 1): Body length 6.89–8.38 mm, width at midbody 0.16–0.20 mm. Anterior half of cephalic vesicle 0.05–0.08 mm long and 0.11–0.13 mm wide; posterior half, 0.06–0.08 mm long and 0.01–0.02 mm wide. Nerve ring, excretory pore, and deirids 0.21–0.28 mm, 0.31–0.36 mm and 0.35–0.41 mm, respectively, from anterior end. Length of esophagus, 0.43–0.56 mm long and 0.068–0.08 mm at its widest region, close to junction with intestine. Synlophe starts immediately behind cephalic vesicle and ends near bursa; 32 ridges at level of nerve ring, 32 at midbody, and 28 near posterior end (Figs. 21–23). Subsymmetric copulatory bursa features a 3 – 2 arrangement and a prominent dorsal ray (Fig. 10); rays 2 to 4 independent, separated at base, and slightly directed anteriad; rays 5 and 6 connected at base, extend laterally with tips that emerge from margin; dorsal ray bifurcates at basis and features a prominent ray 8; rays 9 and 10, subterminal (Fig. 10). Cuneiform spicules, of equal shape and size, 0.12–0.15 mm long, with lamina divided into its middle portion ending at two terminal points, thick lamina dorsally developed, thin vellum extends into proximal part (Figs. 6, 7). Gubernaculum 0.030–0.037 mm long (Fig. 8). Ranges for these values are included in Table 1.

**Table 1.** Comparative measurements of key diagnostic traits of *Torrestrongylus torrei* and *T*. *tetradorsalis*. All measurements are in millimeters.

Measurements		*Torrestrongylus torrei*		*Torrestrongylus tetradorsalis*	
		♀	♂	♀	♂
Length of body		6.2	5.6	7.6–9.2	6.9–8.4
Maximum width				0.17–0.23	0.160–0.200
Cephalic vesicle	Length		0.056	0.060–0.088	0.050–0.080
	Width		0.096	0.130–0.160	0.0.110–0.130
Cervical alae	Length	0.096		0.050–0.090	0.060–0.080
	Width	0.026–0.027		0.010–0.020	0.010–0.020
Nerve ring			0.160	0.260–0.350	0.210–0.280
Excretory pore				0.310–0.400	0.310–0.360
Deirids				0.330–0.440	0.350–0.410
Length of esophagus				0.570–0.680	0.430–0.560
Vulva to anterior end		4.8	–	5.31–6.45	–
Eggs	Length	0.067		0.081–0.0110	
	Width	0.027		0.070–0.080	–
Tail				0.060–0.093	–
Length of terminal spines		0.040			
Dorsal ray			Double bifurcation plus unpaired end		Double bifurcation
Spicules			0.147	–	0.120–0.150
			Trifurcated	–	Bifurcated

**Female** (based on 10 specimens; ranges, see Table 1): Body length 7.45–9.23 mm and width 0.17–0.23 mm. Anterior part of the cephalic vesicle 0.06–0.08 mm long and 0.13–0.16 mm wide, posterior part 0.05–0.09 mm long and 0.01–0.02 mm wide. Nerve ring, excretory pore, and deirids 0.26–0.35 mm, 0.31–0.40 mm, and 0.33–0.44 mm, respectively, from the anterior end. Length of esophagus, 0.57–0.68 mm long and 0.08–0.10 mm wide. Synlophe ridges start immediately behind cephalic vesicle and at basis of terminus and caudal tubercles; 32 ridges at level of nerve ring, 33 at midbody, and 21 at level of posterior end of intestine, close to anus. Amphidelphic. Postequatorial vulva located 5.31–6.45 mm from anterior end; a small extension in the form of flap present in anterior margin of vulva (Figs. 4, 15, 16). Vulva connected to short vagina, leads to vestibule; sphincters short, infundibula elongated, leading to uteri (Fig. 4). Subterminal anus 0.06–0.093 mm from posterior end (Fig. 19). Tail conical. Posterior end includes three tubercles or cuticular processes of various sizes; the largest is dorsal, two of ventral medium-sized, and finally, a thin ventral projection or terminus (Figs. 12, 19, 20). Eggs 0.081–0.11 mm long and 0.07–0.08 mm wide; embryonated (Fig. 9). Ranges for these values are included in Table 1.

### Remarks

The configuration of the cephalic vesicle acts as a reliable character to help discriminate genera in Anoplostrongylinae. *Torrestrongylus torrei* Pérez-Vigueras, 1935 is characterized by the presence of a cephalic vesicle divided into two parts; the anterior has the shape of an umbrella and the posterior is slightly widened, and it has the appearance of “handles of a pitcher” [11]. Based on the structure of the caudal bursa and synlophe, as well as the structure of the cephalic vesicle, the species described herein has been placed in the genus *Torrestrongylus*. The configuration of the cephalic vesicle is markedly different from the homologous structure in other genera in the subfamily, which in most cases possess ornate and simple cephalic vesicles.

The specimens described herein show remarkable similarities to *T. torrei*, especially in the configuration of the cephalic vesicle, the presence of a vulvar flap, and the 3 – 2 bursal arrangement. In addition, the specimens used in the present description were collected from the same species of bat, *Macrotus waterhousii*. *Torrestrongylus tetradorsalis*, however, can be discriminated from *T. torrei* by the absence of cervical alae in both sexes, and by the absence of a terminal unpaired end in the dorsal ray, as seen in *T. torrei* (Plate 7 in [11]). Other differences between these two species include: the relatively shorter size of the posterior half of the cephalic cap of *T. torrei*; the general shape and distal tip of spicules (trifurcated in *T. torrei* vs. bifurcated in *T. tetradorsalis*); the larger body size of specimens of *T. tetradorsalis*; the location of the nerve ring relative to the anterior end, and the larger size of eggs in *T. tetradorsalis* (Table 1).

### Phylogeny

**Figure 27.**
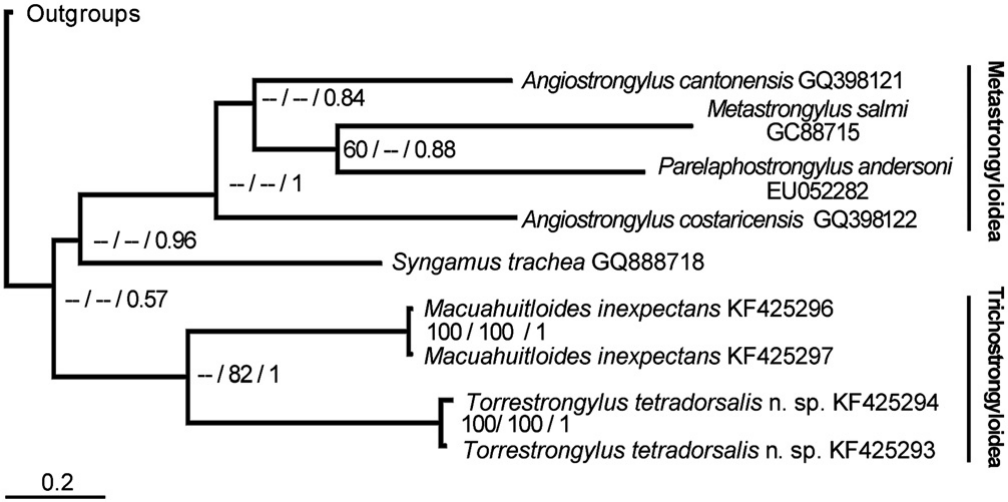
Relative position of *Torrestrongylus tetradorsalis* and *Macuahuitloides inexpectans* as reconstructed in the ML tree used to estimate the posterior probabilities of the branches. The numbers to the right of each node indicate bootstrap support for parsimony and maximum likelihood as optimality criteria and the posterior probability of the branch. *Syngamus trachea* belongs to Strongyloidea. The tree and data matrix can be retrieved from http://opensiuc.lib.siu.edu/zool_data/4/

According to sequences of available taxa, the genetic difference between the two specimens of *T. tetradorsalis* is 0.5%; the posterior credibility of the clade *T. tetradorsalis* + *M. inexpectans* is 1. This clade has moderate bootstrap support using maximum likelihood as an optimality criterion, but no support using parsimony (Fig. 27).

## Discussion

Other members of Anoplostrongylinae that show similarities to *T. tetradorsalis* include *Anoplostrongylus paradoxus* (Travassos, 1918) Boulenger, 1926 and some species of *Tricholeiperia* Travassos, 1935 [20]. However, *T*. *tetradorsalis* cannot be placed in these genera because of the configuration of the cephalic vesicle, caudal bursa, and structure of the synlophe. On the one hand, when compared with *A. paradoxus*, *T. tetradorsalis* shows a prominent dorsal ray, more complex spicules, and a relatively equal length of branches of posterior and anterior vestibules. On the other hand, species in *Tricholeiperia* feature an umbrella-shaped cephalic vesicle, and only *Tricholeiperia poeyi* Baruš and Valle, 1967 features a divided cephalic vesicle [2]. The cephalic vesicle of this species, however, appears to be formed by an anterior dome-shaped half and a widened posterior half. This configuration is markedly different to the cephalic vesicle present in the other species of the genus [5, 9, 19, 21], which feature a vesicle barely separated from the rest of the body [9, 19], or conspicuous expansions divided into two lateral halves [21].

The main difference between species of *Tricholeiperia* and *Torrestrongylus* is the configuration of the cephalic cap. In the present study, one of the specimens (GenBank Accession Numbers KF425295, HWML68494) features a poorly developed posterior half of the cephalic vesicle, which suggests it belongs to *Tricholeiperia*. However, the genetic divergence with *T. tetradorsalis* (GenBank Accession Numbers KF425294, HWML68493) is 0.5%, which suggests they belong to the same species. These results show the urgent need for an extensive phylogenetic analysis including other Anoplostrongylinae featuring the umbrella cephalic vesicle, that may allow the evaluation and proposition of reliable characters used in their diagnosis.

Currently, the list of parasitic helminths of *M. waterhousii* includes five species, two digenetic trematodes including *Limatulum aberrans* Caballero and Bravo, 1950 and *L. oklahomense* Macy, 1931, and three species of nematodes, *Aonchotheca viguerasi* (Freitas and Lent, 1937), *Litomosoides leonilavazquezae* Caballero, 1939 and *Torrestrongylus torrei* [3, 4, 11]. The only species of parasite recorded on *M. californicus* is the nematode *Glyptostrongylus collaris* [22]; however, this is considered a *nomen nudum* since no description is associated with the name. Interestingly, the Waterhouse’s leaf-nosed bats have a disjunct distribution, with a continental cluster localized in western Mexico and north-central Guatemala, and a Caribbean cluster that spans several islands in the Caribbean. *Torrestrongylus torrei* was described from bats in La Habana, Cuba, and *T. tetradorsalis* was collected in bats from Mexico; therefore, these two localities represent both clusters. *Torrestrongylus torrei* is known to infect phyllostomid and molossid bats [11, 14], which suggests this species may be a generalist. The results of our present investigations suggest that in central Mexico, *T. tetradorsalis* is restricted to the Waterhouse’s leaf-nosed bat. However, the expansion of the survey to other areas in the distribution of this species of chiropteran, as well as the inclusion of additional species from other families, will help in establishing the host spectrum for *T. tetradorsalis*. Additional specimens from an unidentified species of *Torrestrongylus* have been reported in two species of phyllostomids in Colombia (*Micronycteris schmidtorum* and *Sturnira bidens*) [6]. An examination of those specimens should allow an evaluation of the variation of the characters used in the diagnosis of species in this genus, and in uncovering the diversity of Anoplostrongylinae in Neotropical bats.
